# Investigation of the Therapeutic Effect of Doxorubicin Combined With Focused Shockwave on Glioblastoma

**DOI:** 10.3389/fonc.2021.711088

**Published:** 2021-07-28

**Authors:** Wei-Hao Liao, Ming-Yen Hsiao, Yi Kung, Abel Po-Hao Huang, Wen-Shiang Chen

**Affiliations:** ^1^Department of Physical Medicine and Rehabilitation, National Taiwan University Hospital and National Taiwan University College of Medicine, Taipei, Taiwan; ^2^Department of Surgery, National Taiwan University Hospital and College of Medicine, Taipei, Taiwan; ^3^Institute of Biomedical Engineering and Nanomedicine, National Health Research Institutes, Miaoli, Taiwan

**Keywords:** glioblastoma multiforme, shockwave, blood-brain barrier, doxorubicin, BBB

## Abstract

**Background:**

Glioblastoma multiforme (GBM) is currently the most devastating brain tumor globally and produces a high mortality rate. GBM is also challenging to eradicate using surgery due to its invasive characteristics. Moreover, the blood-brain barrier (BBB) increases the difficulty of transporting most therapeutic drugs to tumor sites. The use of transcranial focused ultrasound (FUS) has recently been investigated for opening the BBB to facilitate drug delivery. A special form of FUS, the shockwave (SW), has also been shown to open BBB efficiently. SW has several advantages including no heating effect, less reactive oxygen species production, good transcranial ability, and no need to supply microbubbles.

**Methods:**

We employed a commercial SW device, which is a common tool used for musculoskeletal disorders, to improve doxorubicin delivery across the BBB and evaluated its therapeutic efficacy on GBM rat models. SW emits relatively short but stronger mechanical pulses comparing with FUS.

**Results:**

The results demonstrated that doxorubicin combined with SW treatment substantially inhibited tumor growth and prolonged overall survival.

**Conclusions:**

The present study shows the non-invasive transcranial SW may have potential for the treatment of GBM in future clinical setting.

## Introduction

Glioblastoma multiforme (GBM) is the most common and aggressive cancer beginning within the brain. The average incidence of GBM is 3.19 cases per 100,000 persons, and a higher incidence is observed in males and individuals who are Caucasian and non-Hispanic (1). Current treatments of GBM mainly constitute surgery, accompanied by chemotherapy (e.g., temozolomide) and radiation therapy (2–5). However, the effectiveness of these treatments is limited due to the blood-brain barrier (BBB), high heterogeneity of tumor cells, and high toxicity (6). The median survival is only approximately 15-20 months after surgical resection and aggressive chemotherapy together with focal radiotherapy (7). To enhance the therapeutic effect of chemotherapy on GBM, increasing the BBB-crossing capacity is urgently needed for improved treatment of GBM.

Doxorubicin is the most widely used chemotherapeutic agent for cancers. However, it is relatively ineffective for central nervous system tumors due to its poor penetration through the BBB and rapid elimination from the brain tissue by active transport (8). Generally, ultrasound-induced ultrasound contrast agent (UCA) oscillations increase permeability of the blood vessels, thereby promoting drugs to enter the brain parenchyma through the BBB (9).

Recent years pulsed focused ultrasound with UCA has been shown to successfully disrupt the BBB through cavitation in a non-invasive manner (10–12). Several animal studies discovered that doxorubicin delivery to GBM was increased by using focused ultrasound combined with UCA, and found that tumor size was decreased and overall survival was prolonged (13–16). A preliminary clinical study also showed successful delivery of liposomal doxorubicin to brain tissue of GBM patients using MR-guided focused ultrasound, without clinically significant adverse effects (17).

However, focused ultrasound therapy may lead to subtle brain damage by inducing sterile inflammation (18) or neuronal function changes (19). Millions of ultrasonic pulses are administered during focused ultrasound therapy. Although the repeated reflections of acoustic waves were within the skull, and the attenuation of acoustic energy across the skull-brain tissue interface was weak, they may induce unexpected deleterious effects on brain function (20). In addition, the combination of ultrasound and UCA has been demonstrated to increase the production of reactive oxygen species (ROS), and then apoptosis was induced through a caspase-mediated pathway (21, 22). Intracranial hemorrhage also remains a risk under the treatment of focused ultrasound combined with UCAs due to its relatively higher pressure level, huge number of pulses, and much longer treatment duration (23–25).

Our previous animal study discovered a novel BBB-opening system, the shockwave, which demonstrates a general advancement over currently used methods to treat GBM. Compared with traditional focused ultrasound approach, the SW constitutes a form of ultrasonic wave with relatively higher pressure amplitudes, but with much lower number of pulses (26). Its lower frequency characteristic achieved not only a better skull penetration but also avoiding the use of UCA, leading to less ROS production. In addition, SW devices have been commercially available in the treatment of musculoskeletal disorders (27). In the current study, we used SW to enhance the BBB-crossing capacity of doxorubicin and investigated its therapeutic effects on rats with GBM.

## Methods

### Animals

All animal experimental procedures were conducted in accordance with the Care and Use Guidelines of the Laboratory Animal Center at the National Taiwan University College of Medicine, and were approved by the Institutional Animal Care and Use Committee (IACUC, approval no. 20180238) of the National Taiwan University College of Medicine. Fifty-five (55) male Sprague–Dawley rats (8-weeks-old with a body weight of 250‒300 g), purchased from BioLASCO Taiwan Co., Ltd. (Taipei, Taiwan), were used in this study.

For the survival study, the glioma-bearing rats were sacrificed according euthanasia guidelines. If the tumor-bearing animal behavior has the following characteristics such as weight loss more than 20% of the original weight, loss of appetite, weakness, paralysis, head tilt, etc., it will be euthanized.

### Glioma-Bearing Model

We maintained the rat C6-luciferase glioma cell line (C6-Luc), which was transfected with the luciferase gene (luc), according to extant literature (28), and tumor cells were implanted in the rats according to the following modified procedure (28–31): (1) the rats were anesthetized using 3% isoflurane in oxygen; (2) the caudoputamen of each rat brain (0.5 mm anterior and 2.0 mm lateral to the bregma; 5 mm deep) was stereotactically injected with 5 × 10^5^ C6-luciferase cells using a Hamilton syringe within 2 min; and (3) the skull hole was sealed with bone wax, and the wound was rinsed with iodinated alcohol. The glioma-bearing rats were fed post-implantation until sacrifice.

### Bioluminescence Imaging

**Figure 1:**
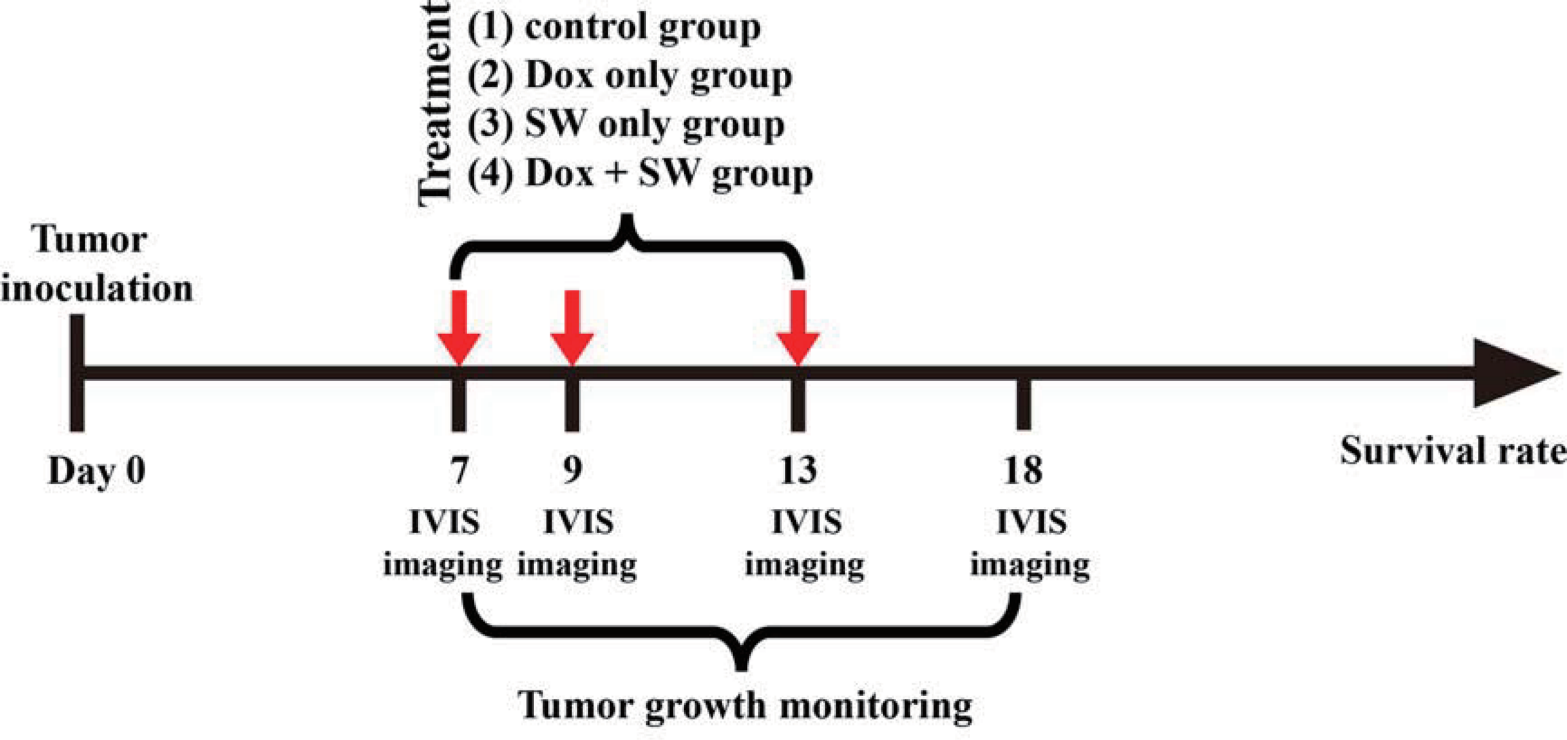
Experimental timeline for the treatment. SW, shockwave; Dox, doxorubicin; *in-vivo* imaging system.

Bioluminescence imaging (BLI) is a sensitive and non-invasive technology used for monitoring *in-vivo* cell growth based on luciferase activity (30, 32, 33). We monitored tumor growth prior to the treatment using an *in-vivo* imaging system (IVIS) (Perkin Elmer, Waltham, MA, U.S.A.). In order to use BLI as an indicator of tumor growth accurately, we inoculated C6-Luc cells into brain of five mice (C1 to C5). At day 7, 9, 13, 18, the glioma-bearing rats were injected with 200 μL (100 mg/kg) of D-Luciferin (Biosynth^®^, Berkshire, UK) under anesthesia using 3% isoflurane (**Figure 1**), and were imaged by IVIS using the following parameters: field of view B, 1-min exposure time, medium binning, and f/stop = 1. Luciferase activity was measured using Live Image 2.5 Software (Perkin Elmer, Waltham, MA, U.S.A.). The results were used as the standard curve for further BLI analysis.

### Shockwave Treatment

We induced the BBB opening by setting the UCA-free SW (PiezoWave, Richard Wolf GmbH, Knittlingen, Germany) at an energy dosage of 0.21 mJ/mm^2^ (intensity level 5), a pulse repetition frequency of 5 Hz, and 200 times (approximately 40 seconds), as described in our previous report (26). The SW probe positioning platform and its implementation were previously described in Kung et al.’s study (26). The BBB opening was evaluated using Evans blue extravasation. Two (2) SW-treated and two (2) control rats were intravenously injected with 30 mg/kg of Evans blue (2% in saline), and then they were sacrificed for brain sectioning after 2 h. Representative sections were stained with H&E, and Nissl stain, to evaluate tissue damage.

### Measurement of Doxorubicin Delivery

To measure the concentration of doxorubicin in the brain after using the focused SW, we utilized the normal rat brain and tumor rat brain to investigate the difference between doxorubicin-plus-focused-SW and doxorubicin alone. Ten (10) normal rats and ten (10) glioma-bearing rats were separately divided into two groups to receive doxorubicin-plus-focused-SW or doxorubicin alone. The contralateral brain of glioma-bearing rats (i.e., the contrast of tumor-bearing site) served as the control.

The doxorubicin-treated rats were sacrificed at 2 h after the focused SW treatment. The harvested brains were washed by cardiac perfusion to remove the unabsorbed doxorubicin. The samples were homogenized in 20X volumes (volume/weight tissue) of acidified ethanol, and refrigerated overnight at 4°C. Subsequently, the samples were centrifuged at 16,000 × g for 25 min at 4°C, and then the supernatant was collected for fluorometric assay. The fluorescent intensity of the supernatant was measured using a microplate reader at an excitation/emission of 480 nm/590 nm (Infinite M200, Tecan, Zurich, Switzerland). The detected doxorubicin was quantified using a linear regression and a standard curve derived from eight serial concentrations of doxorubicin.

### Anti-Glioma Efficacy

The C6-Luc glioma-bearing rat models were established as described above. Glioma-bearing rats were randomly divided into four groups 7 days after the implantation of tumor cells. The first group was the control group, which did not receive doxorubicin and focused SW (control group; n = 5). In the second group, the rats were only intravenously administered with doxorubicin (3 mg/kg on day 7, 9, 13) (Dox group; n = 5). In the third group, the rats were only treated with focused SW (C + SW; n = 4). In the fourth group, rats waited 5 minutes after administration of doxorubicin (3 mg/kg on day 7, 9, 13) before receiving focused SW treatment (Dox + SW group; n = 5). **Figure 1** shows the experimental timeline for different treatments. A total of 19 mice above were used to analyze tumor growth and survival rates. To monitor the tumor progression, the BLI were measured at different time intervals (**Figure 1**) to analyze tumor growth rate (relative to day 7).

The survival times of animals were recorded, and the data were processed by Kaplan–Meier survival log-rank analysis to evaluate the anti-glioma efficacy of different treatments. The end point of the experimental animals was performed in accordance with the above-mentioned euthanasia guidelines. In addition, three mice in each group (total 12 mice) were used for hematoxylin and eosin staining (H&E) to observe tumor size (20 days after C6-glioma inoculation).

### Statistical Analysis

Continuous variables were summarized using number, mean, and standard deviation (SD). The comparison between groups was conducted by one-way ANOVA with LSD *post-hoc* test. The survival status was analyzed using the Kaplan-Meier method. A significant difference was defined as *p* < 0.05. Data analyses were performed using statistical analysis software (SPSS) version 12.0 (IBM, Armonk, NY, U.S.A.).

## Results

### The Concentration of Doxorubicin in the Rat Brain Was Increased Under Shockwave Treatment Through the BBB Opening

**Figure 2:**
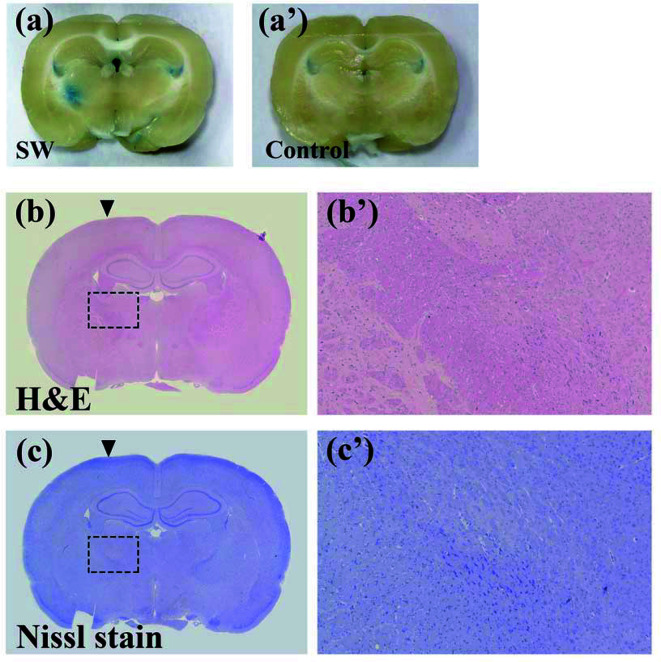
SW treatments inducing the blood-brain barrier (BBB) opening did not damage brain tissue. The BBB opening was evaluated using Evans blue extravasation (**A** and a’). Brain damage in rats with or without SW treatment was assessed using H&E staining (**B** and b’) and Nissl staining (**C** and c’). SW, shockwave.

**Figure 3:**
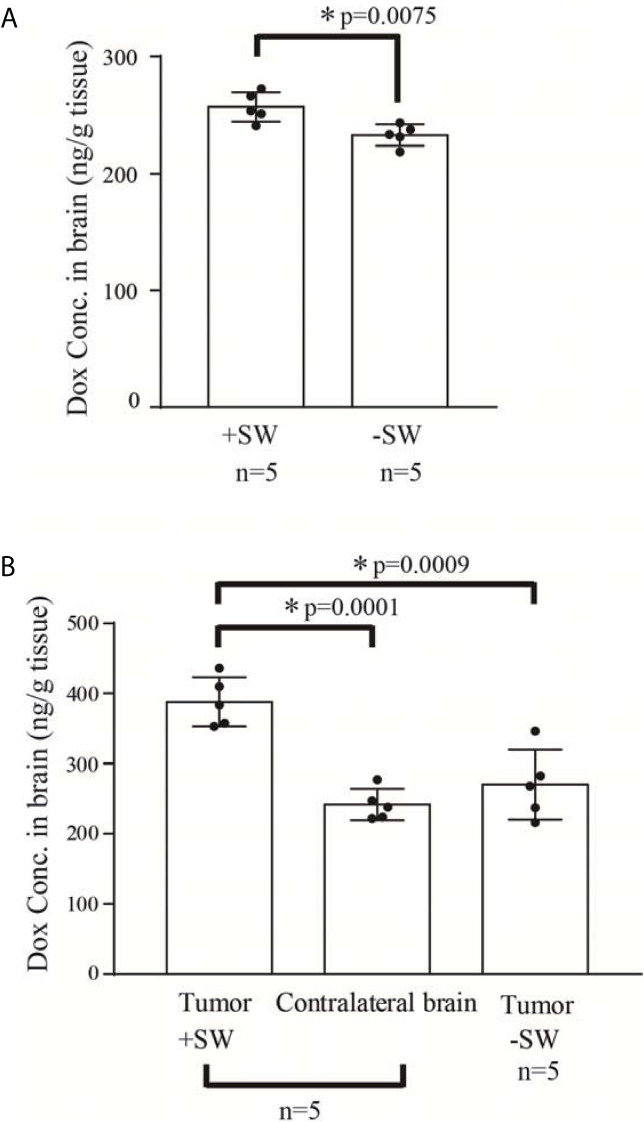
Concentration of doxorubicin in the rat brain. **(A)** Concentration of doxorubicin in the normal rat brain with or without SW treatment (n = 5 for each); **(B)** concentration of doxorubicin in the C6 glioma-bearing brain with or without SW treatment and contralateral brain (n = 5 for each). *P*-value < 0.05 is considered as a significant difference between groups and summarized with an asterisk (*). SW, shockwave; Dox, doxorubicin.

Our previous studies proved that SW effectively induces BBB opening without UCAs or microbubbles (26, 34). In addition, the current research demonstrates that SW effectively opened the BBB without causing tissue damage (**Figures 2A–C**). Furthermore, we investigated whether SW treatment increased the penetration of doxorubicin into the brain *via* eliciting BBB opening. The concentration of doxorubicin in SW-treated brain tissue was significantly higher than that in non-SW-treated tissue (**Figure 3A**; *p* = 0.0165). Likewise, the level of doxorubicin in SW-treated glioma-bearing rat brain was significantly higher than that in contralateral brain tissue (*p* = 0.0072) and non-SW-treated glioma-bearing rat brain (**Figure 3B**; *p* = 0.0103). These results indicate that SW treatment can significantly enhance delivery of doxorubicin to the brain.

### Antitumor Standard BLI Curve

**Figure 4:**
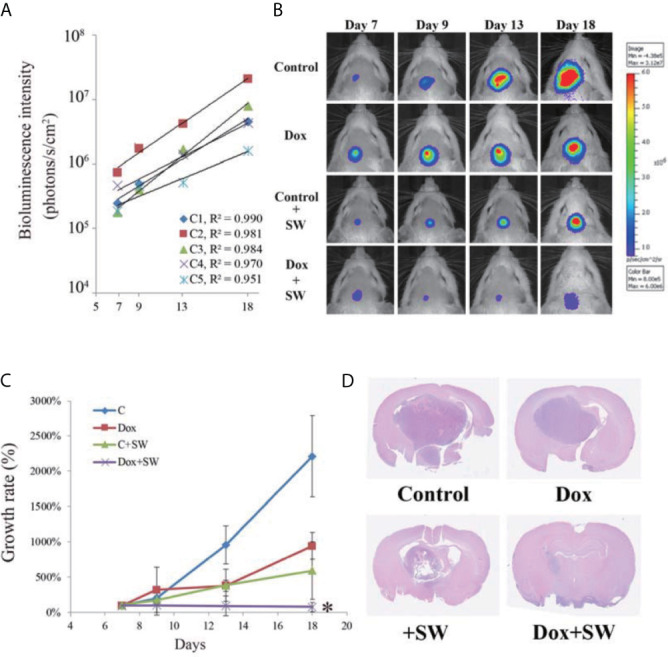
Results of tumor progression from day 7 to day 18. **(A)** The intensity of bioluminescence imaging (BLI) in control group from day 7 to 18 is illustrated using scatter plots. These correlation coefficients show that the intensity of BLI was associated with tumor growth (R2 > 0.95). **(B)** BLI images of each group were collected from day 7 to 18 after tumor cell implantation. **(C)** Tumor growth rate (relative to day 7) on the basis of bioluminescence images. **(D)** Brain tumor size on day 20 after C6-glioma inoculation using H&E staining. An asterisk (*) indicates a statistically significant difference (*p* < 0.05). SW, shockwave; Dox, doxorubicin.

In **Figure 4A**, C1~C5 are the BLI results of five independent mice for the standard curve, showing that BLI provides excellent correlation with tumor growth during day 7 to day 18 (all R^2^ >0.095). Thus, the BLI of the experimental groups could be standardized and compared accordingly.

### Antitumor Effects of Doxorubicin on Tumors Under Shockwave Treatment

The treatment response of doxorubicin combined with SW to GBM was assessed using BLI. Compared with other groups, tumor growth in the Dox + SW group was significantly inhibited on day 18 (**Figures 4B, C** and **Supplementary Figure 1**). Data from H&E staining also showed that tumor size in rats treated with doxorubicin and SW was smaller than that in other groups (**Figure 4D**). These data revealed that doxorubicin combined with SW treatment was able to effectively inhibit tumor growth.

### Prolonged Survival of Glioma-Bearing Rats After Doxorubicin Combined With Shockwave Treatment

**Figure 5:**
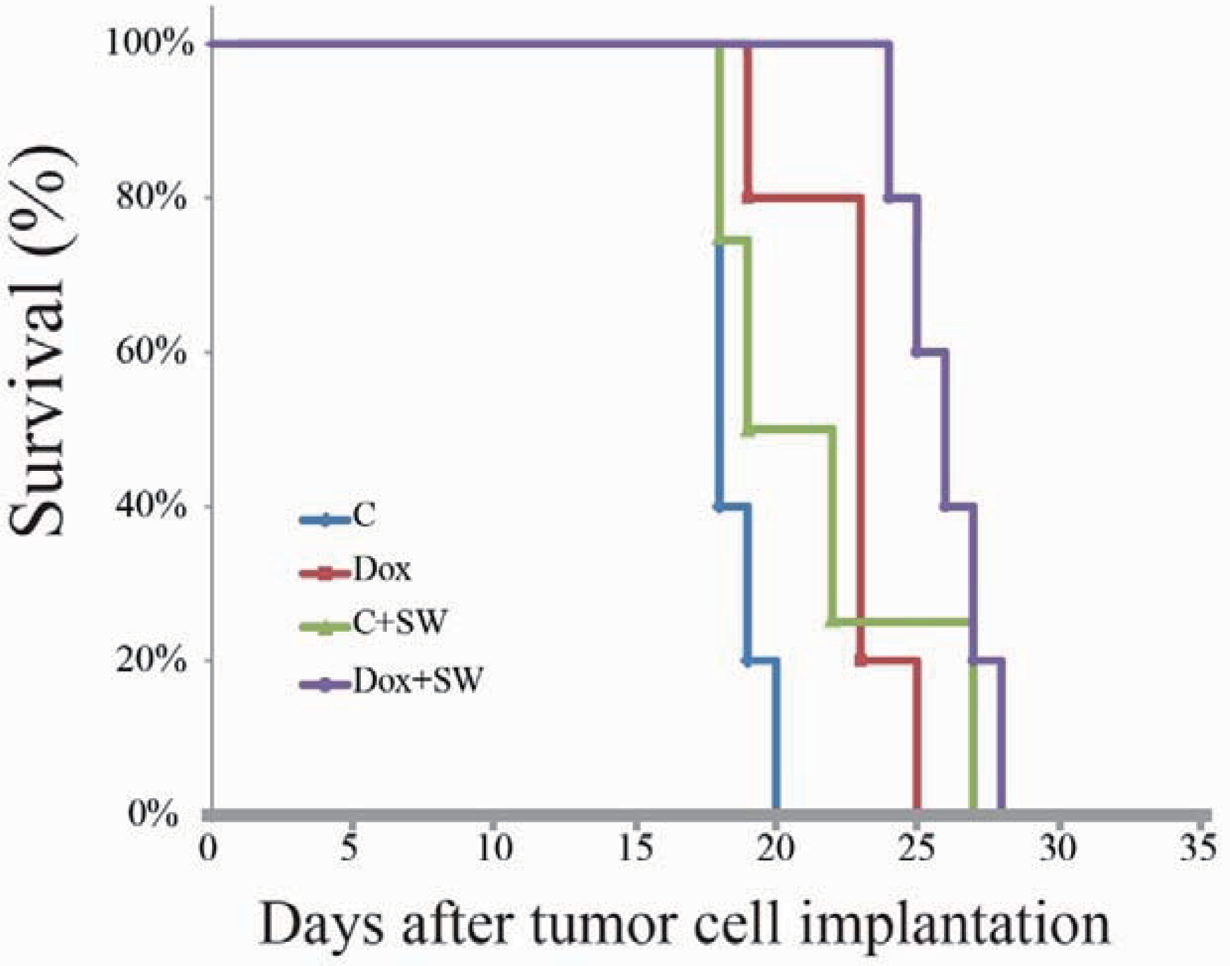
Kaplan-Meier survival curves of C6 tumor-bearing mice treated with different group (Control, Dox, SW, Control +SW and Dox+SW). SW, shockwave; Dox, doxorubicin.

The overall survival of C6-Luc bearing rats receiving doxorubicin and SW was significantly longer than that in rats receiving different regimens, based on the Kaplan-Meier curve (**Figure 5**). The median survival in the Dox + SW group was 26 d, which was longer than the Dox group (23 d), the C + SW group (19 d), and the control group (18 d). Moreover, rats receiving doxorubicin combined with SW had a significantly greater median survival (44.4%) in comparison with control rats (*p* = 0.015), while there was no significant difference between the Dox and control groups (*p* = 0.088). These results indicated that the survival time of dox-treated rats was prolonged under SW treatment due to increased delivery of doxorubicin *via* the BBB opening.

## Discussion

A major contribution of our SW approach is that it not only achieves results that are similar or superior to those of currently used approaches, but it does so with markedly fewer and less serious side effects. Our previous studies have shown that there was almost no generation of ROS (including singlet oxygen, hydroxyl radical and superoxide) through SW treatment, and even if microbubbles were added to promote cavitation, ROS was not increased (23). On the contrary, ultrasound significantly increased ROS. This increased free radical generation can result in apoptosis, cell structure damage, inflammation, DNA disruption, and consequent pathogenesis (35, 36). As mentioned earlier, within 5 min of focused ultrasound exposure, BBB disruption was associated with increased expression of damage-associated molecules leading to a sterile inflammation through the NF-κB pathways (18). One of the major concerns of focused ultrasound sonication is its multiple pulses and longer treatment duration. It is estimated that for one 5 min treatment course, a total of about 150 million pulses will be delivered through skull, before considering the multiple reflections and within the skull. In our SW setting, only 200 pulses were delivered. The brain concentration of doxorubicin after SW treatment was equal or lower than that reported in other studies with focused ultrasound approach (~ 400 ng/g tissue vs. 400–800 ng/g tissue (37, 38). It is probably because UCA was not added during the SW treatment, and the number of pulses were considerably less, so the degree of BBB opening was relatively low compared with other focused ultrasound studies. However, the concentration of doxorubicin seems to be enough, able to effectively inhibit the tumor growth. Other focused ultrasound studies demonstrated the potential of using liposomal doxorubicin to facilitate the drug delivery in the brain parenchyma, reporting remarkably higher tissue concentration, 3–20 μg/g, without obvious side effects (16, 39). The combination of SW and liposomal doxorubicin (or other doxorubicin encapsulation methods) could also be considered if higher brain concentration is necessary.

UCA could also be combined with SW for intracranial drug delivery. Our previous study demonstrated the addition of UCA could effectively reduce the threshold of BBB opening, decrease the necessary pulse number, and enhance opening efficiency (23). However, output pressure should be carefully reduced to avoid possible side effects due to enhance cavitation effect.

To the best of our knowledge, this is the first study to evaluate the effects of doxorubicin combined with SW on GBM. The results show that the new combination therapy is suitable for increasing the delivery of drugs which cannot penetrate the brain due to the BBB. We also observed that doxorubicin combined with SW treatment could effectively suppress tumor growth (**Figure 4**) and prolonged survival time in comparison with doxorubicin-only treatment (**Figure 5**). However, all rats treated with doxorubicin and SW rapidly expired on day 11 after cessation of treatment (i.e., day 24 after tumor inoculation). Our previous *in-vitro* experiments also showed that approximately 30% of C6 glioma cells survived under a high-dose doxorubicin administration, and started to grow after the drug administration was stopped (data not shown). Another possible reason that the therapeutic effects of doxorubicin are limited because the excretion of doxorubicin in cancer cells is driven by efflux transporters (e.g., P-glycoprotein and breast cancer resistance protein), resulting in rapid growth of these surviving cancer cells after drug withdrawal. Recent studies reported that inhibiting the activity or expression of efflux transporters can effectively enhance the effectiveness of doxorubicin in killing cancer cells (40). Moreover, with the same SW delivery approach, we could try other chemotherapeutic agents.

It is worth mentioning that only SW treatment resulted in significant inhibition of tumor growth, but it does not help to improve survival. We speculate that the phenomenon may be due to [1] the cavitation effect produced by SW may directly disrupt the tumor tissue. Because the tumor tissue is not as dense as the intact brain, it is easily damaged by the influence of cavitation. Previous computational study of SW to open the BBB showed that when the SW was applied but there was no bubble, it would not cause damage to the tight junction. However, bubble collapse would destroy the tight junction when the same SW strength was combined with bubbles (41, 42). Although microbubbles were not used in this study to enhance cavitation, there are large amount of gas dissolved in the blood and a large number of blood vessels in the tumor tissue. Therefore, SW greatly enhances the cavitation effect in the tumor, which leads to damage the tumor tissue, affect the nutritional supply (perturb angiogenesis), and ultimately inhibit tumor growth. In fact, there are similar results were reported in the previous focused ultrasound reports (31, 43, 44). [2] The SW may separate the tumor cells so that the bioluminescence signal is not concentrated enough to be captured by IVIS. This may explain why the tumor is small but SW treatment alone cannot effectively improve the survival rate (**Figure 5**). In addition, [3] the SW may increase the risk of tumor metastasis to surrounding tissues, and the simultaneous administration of Dox can reduce this side effect.

The molecular mechanism of using SW to open the blood-brain barrier is not fully understood. In addition to the aforementioned computational studies, our previous research indicated that SW can activate TRPV4 channels on vascular endothelial cells membrane to promote C^2+^ influx into endothelial cells and then activate the PKCdelta signaling pathway, which finally leads to the disruption of tight junctions (34). Adding TRPV4 agonists was shown to reduce the necessary SW intensity, which may help to reduce tissue damage caused by excessive mechanical force of SW in future applications.

### Limitations

There are several limitations to our study. The major limitation was the side effects caused by SW. Although our previous report and this result show that it does not cause significant inflammation and apoptosis, the cavitation produced by SW is much greater than ultrasound, and many people still question its impact on tissues, especially the brain. The mechanism of inducing apoptosis requires further elucidation, such as the expression of caspases and cleaved-caspases need to be observed. In addition, animal behavior analysis can also be considered in the future to illustrate whether SW affects brain function. Second, previous studies reported micrometastasis caused by SW (45). Although the intensity of the SW used in this study is lower than those used in previous studies, this possibility cannot be ruled out. It may explain that SW treatment alone reduced the tumor size, but its survival rate was not improved.

## Conclusion

This animal study sheds light on the treatment of GBM. The focused SW without UCAs, may enhance chemotherapeutic agents to penetrate the BBB, which in turn leads to the suppression of tumor growth and prolongation of overall survival. This method also achieves these results without the deleterious effects presented by currently used methods. However, the potential risks of focused SW to the brain and its clinical applications should continue to be investigated in the future.

## Data Availability Statement

The original contributions presented in the study are included in the article/**Supplementary Material**. Further inquiries can be directed to the corresponding authors.

## Ethics Statement

All animal experimental procedures were conducted in accordance with the Care and Use Guidelines of the Laboratory Animal Center at the National Taiwan University College of Medicine, and were approved by the Institutional Animal Care and Use Committee (IACUC, approval no. 20180238) of the National Taiwan University College of Medicine.

## Author Contributions

W-SC devised the project, the main conceptual ideas, and proof outline. W-HL worked out almost all of the technical details, and performed the experiments,and analyzed the data. M-YH conducted literature review. YK establishes an animal model of the opening of the blood-brain barrier through shockwave treatment. W-HL, AH, and W-SC wrote the manuscript. All authors contributed to the article and approved the submitted version.

## Funding

This study was supported in part by grants from Ministry of Science and Technology (MOST) (grant no. MOST 105-2923-B-002-001-MY3 and 108-2314-B-002-164), and by the National Health Research Institutes (NHRI) (grant no. BN-109-PP-03).

## Conflict of Interest

The authors declare that the research was conducted in the absence of any commercial or financial relationships that could be construed as a potential conflict of interest.

## Publisher’s Note

All claims expressed in this article are solely those of the authors and do not necessarily represent those of their affiliated organizations, or those of the publisher, the editors and the reviewers. Any product that may be evaluated in this article, or claim that may be made by its manufacturer, is not guaranteed or endorsed by the publisher.
